# Classification of Monocytes, Promonocytes and Monoblasts Using Deep Neural Network Models: An Area of Unmet Need in Diagnostic Hematopathology

**DOI:** 10.3390/jcm10112264

**Published:** 2021-05-24

**Authors:** Mazen Osman, Zeynettin Akkus, Dragan Jevremovic, Phuong L. Nguyen, Dana Roh, Aref Al-Kali, Mrinal M. Patnaik, Ahmad Nanaa, Samia Rizk, Mohamed E. Salama

**Affiliations:** 1 Division of Anatomic and Clinical Pathology, Mayo Clinic, Rochester, MN 55905, USA; 2 Division of Cardiovascular Diseases, Mayo Clinic, Rochester, MN 55905, USA; 3 Division of Hematopathology, Mayo Clinic, Rochester, MN 55905, USA; jevremovic.dragan@mayo.edu (D.J.); nguyen.phuong@mayo.edu (P.L.N.); roh.dana@mayo.edu (D.R.); 4 Division of Hematology, Mayo Clinic, Rochester, MN 55905, USA; alkali.aref@mayo.edu (A.A.-K.); Patnaik.Mrinal@mayo.edu (M.M.P.); nanaa.ahmad@mayo.edu (A.N.); 5 Department of Clinical Pathology, Cairo University, 11562 Cairo, Egypt; rizksh@gmail.com

**Keywords:** digital imaging, artificial intelligence, improving diagnosis accuracy, monocytes, promonocytes and monoblasts, chronic myelomonocytic leukemia (CMML) and acute myeloid leukemia (AML) for acute monoblastic leukemia and acute monocytic leukemia, concordance between hematopathologists

## Abstract

The accurate diagnosis of chronic myelomonocytic leukemia (CMML) and acute myeloid leukemia (AML) subtypes with monocytic differentiation relies on the proper identification and quantitation of blast cells and blast-equivalent cells, including promonocytes. This distinction can be quite challenging given the cytomorphologic and immunophenotypic similarities among the monocytic cell precursors. The aim of this study was to assess the performance of convolutional neural networks (CNN) in separating monocytes from their precursors (i.e., promonocytes and monoblasts). We collected digital images of 935 monocytic cells that were blindly reviewed by five experienced morphologists and assigned into three subtypes: monocyte, promonocyte, and blast. The consensus between reviewers was considered as a ground truth reference label for each cell. In order to assess the performance of CNN models, we divided our data into training (70%), validation (10%), and test (20%) datasets, as well as applied fivefold cross validation. The CNN models did not perform well for predicting three monocytic subtypes, but their performance was significantly improved for two subtypes (monocyte vs. promonocytes + blasts). Our findings (1) support the concept that morphologic distinction between monocytic cells of various differentiation level is difficult; (2) suggest that combining blasts and promonocytes into a single category is desirable for improved accuracy; and (3) show that CNN models can reach accuracy comparable to human reviewers (0.78 ± 0.10 vs. 0.86 ± 0.05). As far as we know, this is the first study to separate monocytes from their precursors using CNN.

## 1. Introduction

The classification of the monocytic subpopulations (monoblasts, promonocytes, and monocytes) is important for the proper diagnosis and classification of various monocytic-lineage leukemias, namely, chronic myelomonocytic leukemia (CMML) and acute myeloid leukemia (AML), including acute monoblastic leukemia and acute monocytic leukemia, and acute myelomonocytic leukemia [1].

To meet the World Health Organization (WHO) diagnostic criteria, the peripheral blood (PB) or bone marrow (BM) of patients with acute monoblastic and monocytic leukemia must have ≥20% blasts (including promonocytes), and ≥80% of the leukemic cells must be of monocytic lineage, including monoblasts, promonocytes, and monocytes. Differentiation between acute monoblastic leukemia and acute monocytic leukemia is based on the relative proportions of monoblasts and promonocytes. In acute monoblastic leukemia, the majority of the monocytic cells (≥80%) are monoblasts, whereas in acute monocytic leukemia, the predominant populations are mature monocytes and promonocytes [1,2,3].

The diagnostic criteria for CMML include PB monocytosis (≥1 × 10^9^/L), in which >10% of the PB leukocytes are monocytes. In addition, the PB and BM blast count of <20% of blasts and promonocytes (a blast equivalent cell) must be ascertained [4,5]. Beyond diagnosis, CMML can be stratified into three subcategories based on accurate enumeration of blasts and equivalents (i.e., promonocytes) in the PB and BM. CMML-0: <2% in PB and <5% in BM, CMML-1: 2–4% in PB or 5–9% in BM, CMML-2: 5–19% in PB and 10–19% in BM [2,6].

As seen from the diagnostic criteria listed above, distinction between CMML and AML, and the staging of CMML, depend on accurate differentiation between blast equivalents (monoblasts and promonocytes) and mature monocytes. WHO classification still uses cytomorphology as the gold standard for the definition of blasts. In many cases, the expression of immature marker CD34 is used to supplement the enumeration of blasts. However, monoblasts are frequently negative for CD34 [7], and there are no other reliable immunophenotypic markers to distinguish monoblasts and promonocytes from mature monocytes. As a result, the differential diagnosis in these cases relies solely on cytomorphology.

In general, monocytes are mature cells with minimal morphologic atypia. However, atypical monocytes can be present with abnormal cellular features such as unusually fine chromatin but with prominent nuclear folds or convolutions that partially overlap with more immature forms, including monoblasts and promonocytes [8,9]. This renders distinguishing them from the immature forms notoriously difficult and might lead to under- or overestimation of blast cell numbers [10].

In this article, we present the applicability of artificial intelligence using convolutional neural network architecture for separating monocytes from the spectrum of monocyte precursors (i.e., promonocytes and monoblasts) with reference labels generated based on experts’ morphologic review consensus. Differentiating myeloblasts from monoblasts solely on optical cytology can be very difficult; therefore, we will refer to monoblasts as blasts (monoblasts and/or myeloblasts) in this manuscript.

## 2. Methods

We trained convolutional neural network (CNN) architecture on digital images of monocytes, promonocytes, and blasts to separate monocytes from monocyte precursors (i.e., promonocytes and monoblasts). We experimented and evaluated several data pre-processing configurations and assessed the performance of well-known CNN architecture in order to find the best-performing CNN model and preprocessing strategy for this classification task. The data were imbalanced; therefore, we used the weighted categorical cross entropy loss function (see Equation (1)) to penalize loss for each category during the training [11,12,13]. We used the Adam optimizer [14] and initialized the learning rate to 1 × 10^−4^.

(1) $$L=\frac{1}{n}\overset{n}{\underset{i=1}{\sum }}\overset{m}{\underset{j=1}{\sum }}-y_{ij}log\left({\hat{y}}_{ij}\right)w_{ij}$$

where *n* is number of samples, *m* is number of classes, y is the true labels, $\hat{y}$ is the predicted labels, and $w_{ij}$ is the weighting for each sample of classes. $w_{ij\;}=max\{n_{0}\ldots \;n_{j}\}/n_{j}\;$ is defined to balance the impact of each class in the loss function.

### 2.1. Data Collection

After approval by the Mayo Clinic institution review board (IRB protocol #19-001950), 935 consecutive monocytic cell images were acquired from the PB smear samples of 10 patients diagnosed with AML with monocytic differentiation and CMML using a 100× objective lens under immersion oil using an Olympus BX53 microscope with Olympus DP74 camera to obtain digital images. Each cell was manually cropped by an experienced hematopathologist (M.E.S.) into 200 × 200 pixel images using HyperSnap V7 software (Hyperionics Technology, Murrysville, PA, USA). In order to eliminate the impact of non-relevant background information that might include red blood cells, artifacts, and platelets, a manually segmented mask was provided for each monocytic cell. The cytoplasm and nucleus were labelled separately in each segmentation mask. All collected cells were split into 3 categories (i.e., monocyte, promonocyte, and blast) by 5 hematopathology experts. The consensus between the five experienced morphologists (four hematopathologists, D.J., P.L.N., S.R. and M.E.S., and an experienced pathologist assistant, D.R.) was considered as a ground truth reference label for each cell.

### 2.2. Experiments and Evaluations

We split the data into 70%, 10%, and 20% for training, validation, and testing purposes, respectively, and assessed the performance of five well-known CNNs architectures: InceptionV3 [15,16], Resnet50 [17], Inception_resnet [18], VGG16 [19], and Densenet121 [20]. The training set was used for learning about the data. The validation set was employed to establish the reliability of learning results, and the test set was used to assess the generalizability of a trained model on the data that were not seen by the model. Furthermore, we applied stratified 5-fold cross validation to the best-performing model configuration to further assess the generalization ability of the model. In the 5-fold cross validation, the data were divided randomly into 5 equal sized pieces and samples of each class were equally distributed to each piece. One piece was reserved for assessing the performance of a model, and the remaining 4 pieces were utilized for training models.

**Figure 1:**
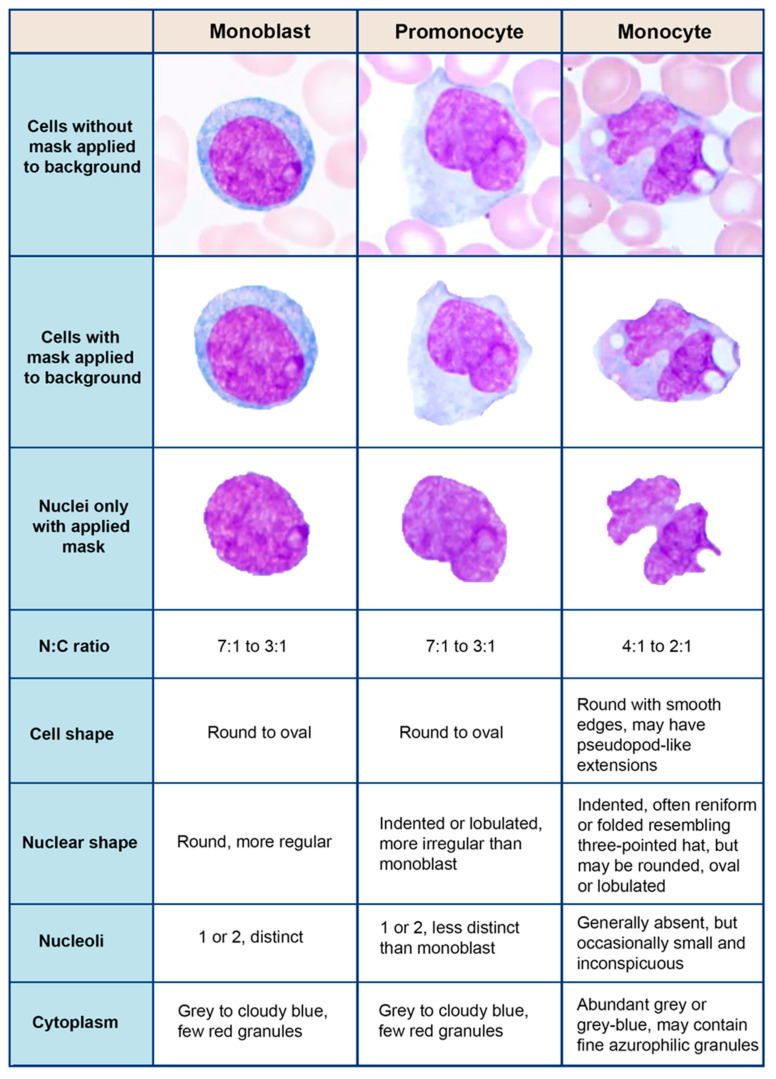
Examples of monocytes, promonocytes, and monoblasts with criteria.

We generated five configurations based on pre-processing input data and assessed the impact of data pre-processing to select the best configuration for our classification task. In configuration 1, cell masks were applied to image patches to suppress the background (i.e., assigning zeros to non-cell pixels) and leave only the cell content in image patches. Afterwards, color normalization (i.e., RGB color channels values were normalized as a percentage of sum of RGB values) was applied to image patches and cells were centered and resized into 200 × 200 pixels. In configuration 2, cell masks were applied to image patches to suppress the background and leave only the cell content in image patches. Next, z-scoring, which is also called the standard score, was applied to image patches. In z-scoring, RGB image channel values were scaled with 0 mean and unit variation. Lastly, cells were centered and resized into 200 × 200 pixels. In configuration 3, image patches without suppressing background (i.e., whole image patched including all the background information) were used as the input data for CNN models. In configuration 4, cell masks were also applied to image patches to suppress the background, leaving only cell content (Figure 1). Lastly, in configuration 5, cell masks were applied to suppress the background as well as the cells of interest but excluding their nuclei, leaving only the nuclei content in image patches (Figure 1). We then centered and resized only the nuclei of each cell into 200 × 200 pixels and applied to them z-scaling to standardize RGB color distribution. For each configuration, we presented accuracy, precision, recall, and F1-score metrics. In addition, we also generated t-SNE plots using the features of the last convolution layer of the best model to show the separation of monocytic cells on the test dataset.

In order to assess the inter-reviewer variability (i.e., the variability between the five expert reviewers), we compared the labels of each reviewer to consensus labels and the average performance and standard deviation were presented. Similarly, to assess the intra-reviewer variability, reviewer 5 labeled the cells a second time (one month later) and a correlation matrix was calculated, as shown in the results section below.

## 3. Results

The performance of the five CNN models with different configurations and the resulting classification of the monocytic cells (i.e., monocyte, promonocyte, and blast) on the validation and test datasets are shown in Table 1 and Table 2. Table 1 shows the results of CNN models with configurations 1–5 for the three-subcategory classification (monocyte vs. promonocyte vs. blast), while Table 2 shows results of CNN models with configurations 1–5 for the two-subcategory classification (monocyte vs. blast + promonocyte). Overall, the Inception_resnet model [18], which is a version of the inception model with residual connection, using configuration 2, gave the best performance in terms of accuracy, precision, recall, and F1-score in the validation and test datasets of both the two-subcategory and the three-subcategory classifications. Densenet121 using configuration 2 was the second-best performing model.

*Color Key for Tables 1–5:*
Relatively Lower Performance        Relatively Higher Performance


**Table 1:** Performance of CNN models using five pre-processing configurations on 3-subcategory (monocytes, promonocytes, and blasts) classification task.

CNN Models	Validation Dataset				Test Dataset			
	Accuracy	Precision	Recall	F1-Score	Accuracy	Precision	Recall	F1-Score
**Configuration 1** (Centered and resized whole cell only and color normalization—cell mask applied)								
Inception_resnet	0.67	0.41	0.64	0.50	0.41	0.36	0.48	0.33
InceptionV3	0.33	0.43	0.41	0.30	0.49	0.46	0.53	0.39
Resnet50	0.62	0.69	0.52	0.50	0.55	0.47	0.49	0.42
VGG16	0.63	0.59	0.68	0.60	0.57	0.54	0.62	0.51
Densenet121	0.68	0.42	0.67	0.51	0.42	0.39	0.50	0.34
**Configuration 2** (Centered and resized whole cell only and z-score pre-processing—cell mask applied)								
Inception_resnet	0.81	0.83	0.80	0.76	0.53	0.50	0.58	0.45
InceptionV3	0.63	0.73	0.62	0.48	0.42	0.36	0.47	0.33
Resnet50	0.63	0.55	0.65	0.56	0.49	0.53	0.56	0.44
VGG16	0.69	0.67	0.74	0.69	0.50	0.54	0.57	0.46
Densenet121	0.72	0.81	0.71	0.63	0.58	0.40	0.60	0.44
**Configuration 3** (Image patch including monocytic cell and surrounding red blood cells—no cell mask applied)								
Inception_resnet	0.71	0.70	0.70	0.59	0.45	0.41	0.52	0.36
**Configuration 4** (Only whole cell presented after applying cell mask)								
Inception_resnet	0.73	0.71	0.73	0.64	0.44	0.41	0.51	0.35
**Configuration 5** (Centered and resized nucleus only and z-score pre-processing—mask applied excluding nucleus)								
Inception_resnet	0.74	0.73	0.76	0.74	0.66	0.65	0.70	0.62

Table 1 shows the Inception_resnet model using configuration 2 performing the best in terms of accuracy, precision, recall, and F1-score in the validation and test datasets of the 3-subcategory classification.

**Table 2:** Performance of CNN models using five pre-processing configurations on 2-subcategory (monocytes and promonocytes + blasts) classification task.

CNN Models	Validation Dataset				Test Dataset			
	Accuracy	Precision	Recall	F1-Score	Accuracy	Precision	Recall	F1-Score
**Configuration 1** (Centered and resized whole cell only and color normalization—cell mask applied)								
Inception_resnet	0.84	0.88	0.83	0.83	0.70	0.75	0.71	0.69
InceptionV3	0.46	0.46	0.46	0.45	0.63	0.63	0.63	0.63
Resnet50	0.63	0.79	0.61	0.55	0.66	0.68	0.65	0.64
VGG16	0.76	0.76	0.76	0.76	0.79	0.82	0.80	0.79
Densenet121	0.87	0.90	0.87	0.87	0.72	0.79	0.73	0.71
**Configuration 2** (Centered and resized whole cell only and z-score pre-processing—cell mask applied)								
Inception_resnet	0.88	0.91	0.88	0.88	0.80	0.83	0.81	0.80
InceptionV3	0.87	0.87	0.87	0.87	0.70	0.74	0.71	0.70
Resnet50	0.80	0.80	0.80	0.80	0.76	0.83	0.77	0.75
VGG16	0.79	0.79	0.79	0.79	0.76	0.83	0.77	0.75
Densenet121	0.79	0.86	0.78	0.77	0.85	0.85	0.85	0.85
**Configuration 3** (Image patch including monocytic cell and surrounding red blood cells—no cell mask applied)								
Inception_resnet	0.87	0.89	0.87	0.87	0.77	0.84	0.78	0.76
**Configuration 4** (Only whole cell presented after applying cell mask)								
Inception_resnet	0.91	0.92	0.91	0.91	0.76	0.83	0.77	0.75
**Configuration 5** (Centered and resized nucleus only and z-score pre-processing—mask applied excluding nucleus)								
Inception_resnet	0.79	0.79	0.79	0.79	0.83	0.85	0.83	0.83

Table 2 shows the Inception_resnet model using configuration 2 performing the best in terms of accuracy, precision, recall, and F1-score in the validation and test datasets of the 2-subcategory classification.

Using configuration 2, the accuracy of CNN models for predicting three subcategories (Table 1) on the test dataset ranged from 42% to 58%, while it ranged from 70% to 85% for predicting two subcategories (Table 2). In the three-subcategory classification (Table 1), the Inception_resnet model achieved 81% accuracy in the validation dataset, but its performance dropped to 53% in the test dataset. In the two-subcategory classification (Table 2), the accuracy of CNN models using configuration 2 ranged from 79% to 88% on the validation dataset. Inception_resnet using configuration 2 provided the most consistent performance in the two-subcategory classification as well in terms of accuracy, precision, recall, and F1-score in the validation and test datasets.

In Table 1 and Table 2, CNN models with configuration 1 showed less consistency between validation and test datasets and had worse performance compared to those with configuration 2. The Inception_resnet model using configurations 3 and 4 showed poor performance compared to the model using configuration 2 (Table 1). However, their performance improved with two-subcategory classification (Table 2). The overall performance of Inception_resnet using configuration 5, which included the nucleus only in image patches, was slightly lower than the performance of the best model in both the two-subcategory and three-subcategory classification tasks, as shown in Table 1 and Table 2.

**Figure 2:**
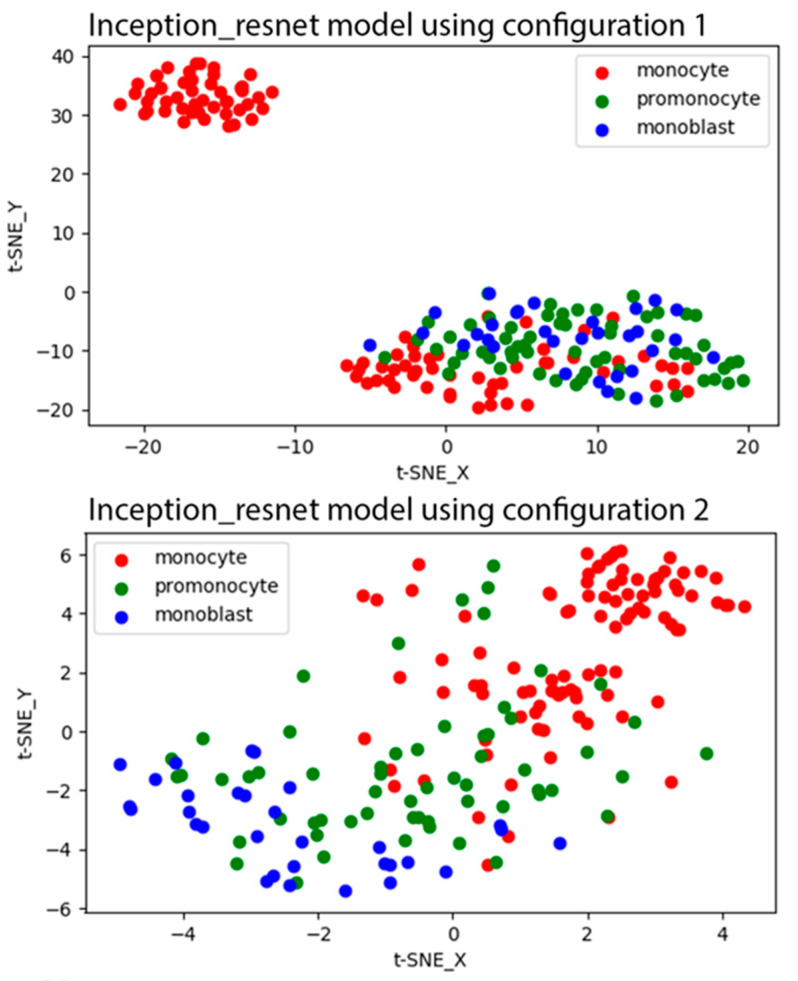
t-SNE plots for the performance of the Inception_resnet model using configurations 1 and 2 on the test. In the configuration 1 plot, all promonocytes demonstrated similar features to blasts and some of monocytes were also not discernable from blasts. In the configuration 2 plot, promonocytes were distributed across monocyte and blast classes.

Figure 2 shows the t-SNE plots for the learned features of the last convolutional layer of the Inception_resnet model with configurations 1 and 2 that were generated from the test dataset. As shown in the t-SNE plot of the Inception_resnet model with configuration 1, all promonocytes demonstrated similar features to blasts, and some of monocytes were also not discernable from blasts. In the t-SNE plot of the model with configuration 2, promonocytes were distributed across monocyte and blast classes. There was a narrow band to differentiate promonocytes from both other classes.

The average performance of the fivefold cross validation using the best performing model, Inception_resnet, is shown in Table 3. The average accuracy of the model and its standard deviation across the fivefold cross validation were 0.66 ± 0.12 and 0.78 ± 0.10 for three-subcategory and two-subcategory classifications, respectively. The performances in the first two iterations, were the lowest while the performance in iteration three was the highest. In the two-subcategory classification, the average performance of the fivefold cross validation (Table 3) was slightly lower than the performance of the Inception_resnet model (Table 2) on the test dataset (78% vs. 80%, respectively).

The performance of the five human expert reviewers compared to the consensus reference labels is shown in Table 4. The mean and standard deviation of the performance of the reviewers were 0.81 ± 0.07 and 0.86 ± 0.05 for the three-subcategory and two-subcategory classifications, respectively. Apart from reviewers 3 and 5, there was a strong consensus between the other three reviewers. The performance of reviewer 3 was 72% accurate, which was the lowest performance among the other reviewers. As seen in Table 4, human performance could be as low as 72% and 80% accurate for the three-subcategory and two-subcategory classifications, respectively. The overall results in the fivefold cross validation test (Table 3) were slightly lower than the human reviewers’ performance in the two-subcategory classification task (0.78 ± 0.10 vs. 0.86 ± 0.05).

A Pearson’s correlation matrix between reviewers and consensus reference labels is displayed in Table 5. The Pearson’s correlation between the five reviewers ranged from 0.5 to 0.75. The correlation between reviewers and consensus reference labels ranged from 0.67 to 0.86. The correlation between the two labels of reviewer 5 (reviewer 5 vs. reviewer 5R) is 0.92 and represents the intra-reviewer variability.

## 4. Discussion

Monocyte assessment is frequently used in day-to-day practice to differentiate neoplastic processes from reactive monocytosis such as infections. According to the WHO criteria, the diagnosis of monocytic neoplasms is dependent on quantitating monoblasts, promonocytes, and monocytes [2]. Specifically, for the accurate recognition and quantification of the two subtypes (promonocytes and monoblasts) most characteristic of acute leukemia, we are required to distinguish between the subtypes of AML with monocytic differentiation and CMML [2,21]. In addition, quantification of monoblasts is necessary for CMML staging, and quantification of monocytes is important for the differential diagnosis of other chronic myeloid neoplasms, including atypical CML [22].

Microscopic evaluation and enumeration of monoblasts, promonocytes, and monocytes by an experienced hematopathologist remains to be the only accepted gold standard; however, morphologic assessment alone can be difficult and subject to significant inter- and intra-observer variability. In fact, monocytes and monocytic precursors are the most difficult cells to identify and classify with confidence in the peripheral blood or in the bone marrow [8]. Other modalities such as multiparameter flow cytometry have been attempted to determine whether immunophenotypic expressions such as anti-CD14 antibodies, which recognize the MO2 and MY4 epitopes, can identify monoblasts, promonocytes, and monocytes [23]. However, the adoption of alternatives to morphology requires technical expertise and remains limited in terms of widespread applicability.

It is imperative that diagnoses distinguish accurately between CMML, including the correct subcategory, and AML with monocytic differentiation, because incorrect diagnosis has significant therapeutic ramifications. For instance, management of CMML is guided by risk categories (high or low risk) based on a CMML-specific scoring system [24] that incorporates the percentage of PB and BM blasts as an important factor determining survival and prognosis [25]. Accordingly, high-risk groups are more subject to hematopoietic cell transplantation—which is associated with significant morbidity and mortality—than the low-risk groups, which are more subject to symptom-directed therapy (e.g., hydroxyurea, hypomethylating agents, and/or supportive care) [26]. Likewise, patients with AML have a different therapeutic approach, because their treatment regimen usually begins with intensive remission–induction chemotherapy, which generally includes a seven-day continuous infusion of cytarabine along with anthracycline treatment on days 1–3 (the so-called “7 + 3” regimen) [27]. This induction therapy can be highly toxic and typically entails hospitalization for several weeks. Hence, precise identification and detailed characterization of monocytic cells is of major relevance not only for diagnosis, but also for treatment. Other neoplastic myeloid conditions have been associated with monocytic abnormalities including juvenile myelomonocytic leukemia, chronic myeloid leukemia with p190 fusion, and myeloid neoplasm with rearrangements of PDGFRA, PDGFRB, FGFR1 and PCM1-JAK2. In addition, monocytosis could be a sign of progression of Philadelphia-negative myeloproliferative neoplasms [28].

The evolution of digital imaging and AI application provides a promising potential in cell-based classification. As such, we thought to evaluate the applicability in monocytic cell-type classification. In this study, we assessed the performance of five well-known CNN architectures for separating monocytes from the spectrum of monocyte precursors (i.e., promonocytes and monoblasts). As mentioned before, ground truth reference labels to train these models were generated based on the consensus of five expert reviewers. Table 4 shows that the percentage of agreement between expert reviewers ranged from 72% to 86% for the three-subcategory classification task, which is a good concordance for such a difficult task. These results were in line with previously reported concordance rates in the literature (76.6%) between expert hematopathologists [8,10]. This agreement was further improved when monocyte precursors were combined. Importantly, consensus on the classification of cells, which is used as the gold standard, was achieved by individual classification of each cell by each one of the evaluators. This is a higher standard than applied in a regular clinical practice, where there are other parameters which could be helpful in reaching the correct percentage of blast-equivalents (for example: similarity between individual cells, bone marrow cellularity, absence of other hematopoietic lineages).

The performance of CNN models did not reach the level of the performance of human experts in separating monocytic cells in the three-subcategory classification, while their performance was significantly improved in the three-subcategory classification, and hence more comparable to the performance of human experts. The improvements in the inter-observer agreement and CNN model support the practice of combining blasts/promonocytes into a single subcategory. As shown in our experiment in the three-subcategory classification (Table 1)—to find the best model and preprocessing approach—we conclude that Inception_resnet using configuration 2 provides the best overall results in validation and test datasets. However, the performance of the other models and configurations, apart from configuration 1, was comparable with small differences in the two-subcategory classification, as shown in Table 2. Even though the results are comparable, configurations using cell masking to suppress the impact of irrelevant background information on the prediction outcome are more reliable. Configuration 5 using nucleus only data also showed consistent results of cross-validation and test datasets, both in the two-subcategory and three-subcategory classifications (Table 1 and Table 2). The impact of the cytoplasm and nucleus on predicting monocytic cells could be further investigated in a larger study to validate our preliminary findings.

The scope of this study was limited to the applicability of monocytic classification based on the morphologic assessment by our expert hematopathology reviewers. Other cell types, immunohistochemical, or flow cytometric immunophenotyping features were not collected to address the reproducibility of the results presented in this article and its direct impact on the diagnosis. Even though we obtained promising results in the identification of monocytes and its precursors using CNNs, these results still need to be validated with a larger study population. We used high-resolution cell images which required the manual acquisition of images. Both image acquisition and cell classification posed challenges that limited the number of cells used in our study.

A larger study with higher numbers of cells could also help further improve the performance of CNN models and obtain a better generalization ability. A larger cohort will likely improve training of the CNN models and could possibly provide an improved ground truth reference. Furthermore, additional work is needed to explore the clinical applicability and clinical validity of such CNN models. Finally, our results underline the fact that monocytic cell differentiation is a difficult task, with relatively low concordance between expert reviewers.

## 5. Conclusions

In summary, we present that CNN models could perform almost as well as human experts in separating monocytes from their precursor cells. To the best of our knowledge, this is the first study to separate monocytes from their precursors using deep learning. Our promising results demonstrate that CNN models could be adopted for this task and further improved with a larger study population.

## Figures and Tables

**Table 3:** Overall performance of fivefold cross validation using the Inception_resnet CNN model.

5-Fold Cross Validation	3-Subcategory (Monocytes vs. Promonocytes vs. Blasts)				2-Subcategory (Monocytes vs. Promonocytes + Blasts)			
	Accuracy	Precision	Recall	F1-Score	Accuracy	Precision	Recall	F1-Score
Iteration 1	0.56	0.60	0.46	0.47	0.67	0.67	0.67	0.67
Iteration 2	0.57	0.55	0.45	0.45	0.68	0.72	0.67	0.66
Iteration 3	0.81	0.79	0.78	0.77	0.89	0.90	0.88	0.89
Iteration 4	0.77	0.75	0.78	0.77	0.83	0.83	0.83	0.83
Iteration 5	0.58	0.56	0.62	0.53	0.81	0.84	0.82	0.81
Mean ± STD	0.66 ± 0.12	0.65 ± 0.11	0.62 ± 0.16	0.60 ± 0.16	0.78 ± 0.10	0.79 ± 0.09	0.77 ± 0.10	0.77 ± 0.10

**Table 4:** Performance of human experts compared to consensus reference labels.

	Reviewers vs. Consensus Reference							
	3-Subcategory (Monocytes vs. Promonocytes vs. Blasts)				2-Subcategory (Monocytes vs. Promonocytes + Blasts)			
	Accuracy	Precision	Recall	F1-Score	Accuracy	Precision	Recall	F1-Score
Reviewer 1	0.86	0.83	0.88	0.85	0.90	0.90	0.90	0.90
Reviewer 2	0.86	0.87	0.84	0.85	0.89	0.89	0.89	0.89
Reviewer 3	0.72	0.77	0.64	0.67	0.80	0.81	0.79	0.79
Reviewer 4	0.86	0.86	0.85	0.85	0.89	0.89	0.88	0.89
Reviewer 5	0.76	0.75	0.80	0.76	0.80	0.82	0.81	0.80
Mean ± STD	0.81 ± 0.07	0.82 ± 0.05	0.80 ± 0.10	0.80 ± 0.08	0.86 ± 0.05	0.86 ± 0.04	0.86 ± 0.05	0.85 ± 0.05

**Table 5:** Pearson’s correlation matrix between reviewers. Reference: consensus of 5 reviewers. Reviewer 5R: second repetition of reviewer 5.

	Reviewer 1	Reviewer 2	Reviewer 3	Reviewer 4	Reviewer 5	Reviewer 5R	Reference
Reviewer 1	1	0.73	0.58	0.75	0.74	0.76	0.86
Reviewer 2	0.73	1	0.61	0.73	0.65	0.66	0.84
Reviewer 3	0.58	0.61	1	0.58	0.5	0.49	0.67
Reviewer 4	0.75	0.73	0.58	1	0.62	0.63	0.86
Reviewer 5	0.74	0.65	0.5	0.62	1	0.92	0.73
Reviewer 5R	0.76	0.66	0.49	0.63	0.92	1	0.73
Reference	0.86	0.84	0.67	0.86	0.73	0.73	1

## Data Availability

The data presented in this study are contained within this article.
